# Computational prediction of interactions between Paxlovid and prescription drugs

**DOI:** 10.1073/pnas.2221857120

**Published:** 2023-03-13

**Authors:** Yeji Kim, Jae Yong Ryu, Hyun Uk Kim, Sang Yup Lee

**Affiliations:** ^a^Metabolic and Biomolecular Engineering National Research Laboratory, Department of Chemical and Biomolecular Engineering (BK21 four), Korea Advanced Institute of Science and Technology, Daejeon 34141, Republic of Korea; ^b^Systems Metabolic Engineering and Systems Healthcare Cross-Generation Collaborative Laboratory, Korea Advanced Institute of Science and Technology, Daejeon 34141, Republic of Korea; ^c^Department of Biotechnology, Duksung Women’s University, Seoul 01369, Republic of Korea; ^d^Systems Biology and Medicine Laboratory, Department of Chemical and Biomolecular Engineering, Korea Advanced Institute of Science and Technology, Daejeon 34141, Republic of Korea; ^e^KAIST Institute for the BioCentury, BioProcess Engineering Research Center, and BioInformatics Research Center, Korea Advanced Institute of Science and Technology, Daejeon 34141, Republic of Korea

**Keywords:** COVID-19, drug interactions, DeepDDI2, Paxlovid

## Abstract

Pfizer’s Paxlovid has recently been approved for the emergency use authorization (EUA) from the US Food and Drug Administration (FDA) for the treatment of mild-to-moderate COVID-19. Drug interactions can be a serious medical problem for COVID-19 patients with underlying medical conditions, such as hypertension and diabetes, who have likely been taking other drugs. Here, we use deep learning to predict potential drug–drug interactions between Paxlovid components (nirmatrelvir and ritonavir) and 2,248 prescription drugs for treating various diseases.

In December 2021, Pfizer’s Paxlovid (nirmatrelvir and ritonavir copackaged for oral use) received Emergency Use Authorization (EUA) from the US Food and Drug Administration (FDA) for the treatment of mild-to-moderate COVID-19 patients. Nirmatrelvir inhibits the severe acute respiratory syndrome coronavirus 2 (SARS-CoV-2) 3C-like protease to prevent virus replication, and ritonavir slows down the degradation of nirmatrelvir by acting as a CYP3A inhibitor. Subsequent clinical studies showed that Paxlovid is effective in reducing the hospitalization risk of COVID-19 patients aged 50 or older (1). A similar result was observed in patients aged 65 or older, who were treated with nirmatrelvir alone (2). Importantly, when the EUA was issued, FDA also provided information on 108 drugs that might exhibit potential drug interactions with Paxlovid (3). Likewise, in January 2022, European Medicines Agency (EMA) also reported 128 drugs that can potentially interact with Paxlovid (‘Paxlovid: EPAR—Product information’ available at: https://www.ema.europa.eu/en/medicines/human/EPAR/paxlovid#product-information-section; Accessed December 22, 2022); FDA and EMA reported 69 drugs in common. Both FDA and EMA reported potentially interacting drugs mainly because these drugs can serve as substrates or inhibitors of cytochromes P450 (CYPs), leading to unwanted drug–drug interactions (DDIs). Such DDIs can be a serious problem for the COVID-19 patients having underlying medical conditions such as hypertension and diabetes because these patients are already taking medicine to treat their conditions (4–7). A problem here is that there are likely more drugs that might interact with Paxlovid, and possible DDIs involving Paxlovid cannot be experimentally examined in a short period of time.

Here, we report the list of a large number of prescription drugs that are predicted to have DDIs with Paxlovid by employing DeepDDI. DeepDDI is a computational model developed using deep learning that predicts the pharmacological effects and adverse drug events (ADEs) of DDIs (8). DeepDDI receives structural information as simplified molecular-input line-entry system of two drugs in a pair as an input, and predicts DDI types as an output in the form of human-readable sentences. The DeepDDI output sentences describe changes in the pharmacological effects and/or the risk of ADEs as a result of the DDI. DeepDDI originally covered a total of 86 DDI types (8), but has been updated in this study to cover 113 DDI types (Fig. 1, Datasets S1 and S2, and *SI Appendix*, *Materials and Methods*). Prediction of potential DDIs with Paxlovid is important for the safety of COVID-19 patients who are already taking other medicines because doctors can prescribe combination of safe drugs by excluding those showing significant ADEs while suggesting alternative prescription drugs with no or minimal ADEs. In this study, the predicted DDIs involving representative prescription drugs were compared with drugs reported to have potential DDIs by FDA and EMA (Fig. 2).

**Fig. 1.**
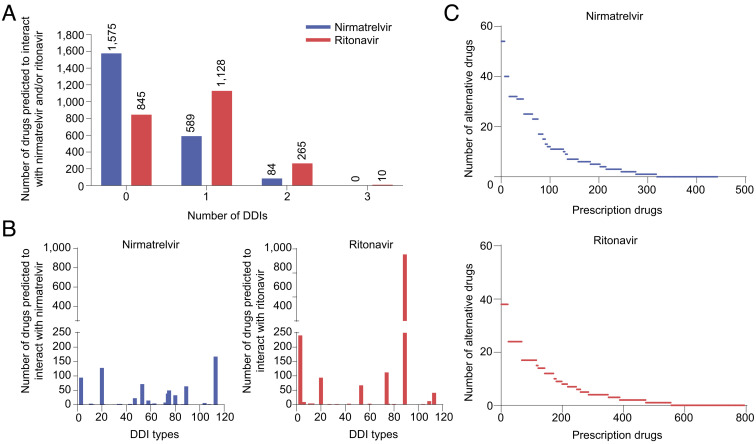
Summary of the DeepDDI2 prediction results between Paxlovid components (nirmatrelvir and ritonavir) and 2,248 prescription drugs. (*A*) Number of DDI types predicted for each prescription drug against nirmatrelvir and/or ritonavir. (*B*) Number of the prescription drugs predicted to interact with nirmatrelvir (*Left* graph) and/or ritonavir (*Right* graph) across the 113 DDI types. Full information is available in Dataset S3. (*C*) Number of alternative drugs for each prescription drug that was predicted to interact with nirmatrelvir (*Upper* graph) and/or ritonavir (*Lower* graph). These alternative drugs were selected if they were predicted to not have any DDIs with nirmatrelvir and ritonavir and if they were reported to have the same mechanism of action as the prescription drug according to Drug Repurposing Hub (https://clue.io/repurposing-app) (9). The details on the prescription drugs and their alternative drugs can be found in Dataset S4. The prescription drugs (numbers) on the X-axis of the upper and lower graphs are presented in the same order as in Dataset S4.

**Fig. 2.**
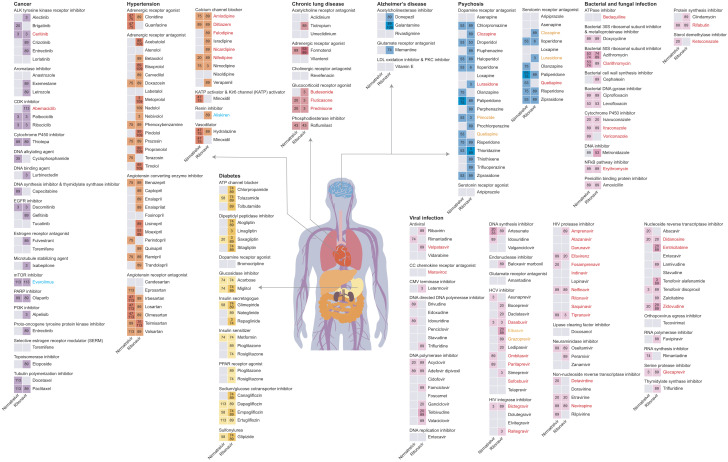
DDI types predicted between Paxlovid components (nirmatrelvir and ritonavir) and representative prescription drugs using DeepDDI2. Colored cells indicate the prescription drugs that were predicted to have DDIs with Paxlovid. Numbers in the colored cells indicate the predicted DDI types. Also, drug names are colored if the DDIs were reported by FDA and/or EMA: red, drugs reported by only FDA; blue, drugs reported by only EMA; orange, drugs reported by both FDA and EMA; and black, drugs not reported by FDA and EMA. Representative keywords associated with each DDI type are: 3, ‘the increased serum concentration of a drug’; 6, ‘the increased serum concentration of the active metabolites of a drug’; 20, ‘the decreased serum concentration of a drug’; 33, ‘the reduced serum concentration of the active metabolites of a drug’; 35, ‘the increased cardiotoxic activities’; 43, ‘the increased immunosuppressive activities’; 47, ‘the increased antihypertensive activities’; 53, ‘the increased QTc-prolonging activities’; 58, ‘the increased hypoglycemic activities’; 74, ‘the decreased therapeutic efficacy’; 75, ‘the increased hypotensive activities’; 89, ‘the decreased metabolism of a drug’; 109, ‘the increased bradycardic activities’; and 113, ‘the increased risk or severity of adverse effects’. The full descriptions of the DDI types can be found in Dataset S2.

## Results and Discussion

Here, we predicted all possible DDIs between Paxlovid components (nirmatrelvir and ritonavir) and 2,248 prescription drugs used for treating diseases using an updated version of DeepDDI (DeepDDI2) that predicts 113 DDI types (Fig. 1, Datasets S1 and S2, and *SI Appendix*, *Materials and Methods*). As a result, 1,628 prescription drugs were predicted to have 2,445 DDIs with nirmatrelvir and/or ritonavir (Dataset S3); specifically, 673 and 1,403 prescription drugs were predicted to interact with nirmatrelvir and ritonavir, respectively. There were no prescription drugs that were predicted to have more than three types of DDIs with nirmatrelvir or ritonavir (Fig. 1*A*). Also, it was predicted that the prescription drugs would interact more with ritonavir than nirmatrelvir (Fig. 1*A*). Among the entire DDI types predicted, DDI type 89 (‘the decreased metabolism of a drug’) was the most frequently predicted one (Fig. 1*B*), which is consistent with a report that Paxlovid is an inhibitor of CYP3A that is associated with drug metabolism (3). In case of hypertension, five calcium channel blockers (i.e., amlodipine, diltiazem, felodipine, nicardipine, and nifedipine) were predicted to have DDIs with the Paxlovid components, which is also consistent with the previous report (3); all the drug combinations involving these five calcium channel blockers were predicted to have DDI type 89 (Fig. 2). Additional DDI types that were frequently predicted include DDI types 3 (‘the increased serum concentration of a drug’), 20 (‘the decreased serum concentration of a drug’), 53 (‘the increased QTc-prolonging activities’), 74 (‘the decreased therapeutic efficacy’), and 113 (‘the increased risk or severity of adverse effects’) (Fig. 1*B*). Of course, these DDI types predicted should be carefully examined by doctors before they prescribe drugs. It should be noted that, among the 108 drugs reported by FDA and the 128 drugs from EMA, 83 and 92 drugs (around 76.9% and 71.9%, respectively) were predicted to potentially interact with at least one component of Paxlovid according to DeepDDI2 (Dataset S3).

Because DeepDDI2 predicts the potential DDIs between the Paxlovid components and the prescription drugs, the prediction outcomes from DeepDDI2 also help finding alternative prescription drugs with no or minimal DDIs (Fig. 1*C* and Dataset S4). In this study, alternative drugs were chosen based on two criteria: 1) They should not be predicted to have any DDI with nirmatrelvir and ritonavir; and 2) they should have the same mechanism of action as the prescription drugs that are predicted to interact with nirmatrelvir and/or ritonavir, as these alternative drugs should also target the same disease. For the second criterion, the mechanisms of action for 445 and 804 drugs, which were predicted to interact with nirmatrelvir and ritonavir, respectively, were found at Drug Repurposing Hub (https://clue.io/repurposing-app; Dataset S4) (9). As an example of the selected alternative drugs, five out of the 49 hypertension drugs (i.e., atenolol, candesartan, fosinopril, labetalol, and nisoldipine) were predicted to have no DDIs with both nirmatrelvir and ritonavir; thus, these hypertension drugs are likely safer options than other hypertension drugs when prescribing Paxlovid (Fig. 2). If a hypertension drug with specific mechanism of action should be prescribed, hypertension drugs that were predicted to have no or fewer DDIs with the Paxlovid components can be considered within the same hypertension drug class; for example, candesartan in place of other angiotensin receptor antagonists, and nisoldipine in place of other calcium channel blockers (Fig. 2). Prescription of an alternative drug, which has the same mechanism of action as an originally prescribed drug, will help minimize ADEs caused by DDIs.

Having the list of 1,628 prescription drugs that were predicted to have DDIs with nirmatrelvir and/or ritonavir and the list of alternative drugs with no or fewer DDIs will help doctors to treat their COVID-19 patients with underlying diseases more safely. However, we would like to emphasize that doctors should not make decisions solely based on these DDI predictions as most of the predictions have not been clinically and/or experimentally validated. In reality, clinical, genetic, and lifestyle factors can impact the occurrence of DDI between multiple drugs (10). Unfortunately, obtaining clinical validation data will take too much time, especially in light of the serious pandemic situation that the world is currently facing. Until such clinical validation data become available, it is hoped that the DDI prediction results presented here will serve as an important resource for physicians to make safer drug prescriptions, after careful analysis and expert consultation, particularly with relevant specialists including pharmacologists and clinical pharmacists, and taking into account all the medically relevant information about the patients. In addition to its potential clinical applications, we hope that the DeepDDI2 prediction results will draw attention to prescription drugs that may have unwanted adverse interactions with Paxlovid, particularly in urgent medical situations like COVID-19, and facilitate further rigorous validation studies for each prediction result.

## Materials and Methods

All the *Materials and Methods* conducted in this study are detailed in *SI Appendix*, *Materials and Methods*: preparation of a gold standard DDI dataset, generation of structural similarity profiles, development of a deep learning model for predicting DDI types (Dataset S5), processing of the initial prediction results from the DNN, preparation of a dataset for prescription drugs from FDA Adverse Event Reporting System, and development environment.

## Supplementary Material

Appendix 01 (PDF)

Dataset 01 (XLSX)

Dataset 02 (XLSX)

Dataset 03 (XLSX)

Dataset 04 (XLSX)

Dataset 05 (XLSX)

## Data Availability

All the data are available in the article and/or SI Appendix. The source code for DeepDDI2 is available at https://bitbucket.org/kaistsystemsbiology/deepddi2 (11).
