# Supplementary material for: Computational prediction of interactions between Paxlovid and prescription drugs
Source: Proc Natl Acad Sci U S A. 2023 Mar 13;120(12):e2221857120. doi: 10.1073/pnas.2221857120 (PMC10041137; doi:10.1073/pnas.2221857120)
Supplement: Supplementary file 1 — Appendix 01 (PDF) [file pnas.2221857120.sapp.pdf]

Supplementary Information for

Computational prediction of interactions between Paxlovid and prescription drugs

Yeji Kim, Jae Yong Ryu, Hyun Uk Kim, Sang Yup Lee

Correspondence should be addressed to: Sang Yup Lee
Email: leesy@kaist.ac.kr

This PDF file includes:

Materials and Methods
SI references

Other supplementary materials for this manuscript include the following:

Dataset S1
Dataset S2
Dataset S3
Dataset S4
Dataset S5

Supporting Appendix SI

Materials and Methods

Preparation of a gold standard DDI dataset

The gold standard DDI dataset for DeepDDI2 was prepared using DrugBank (1) (version 5.1.1) to cover 222,127 DDIs from 222,041 drug pairs (Dataset S1), which represent 113 DDI types (Dataset S2). The newly prepared dataset was split into training (80%) and test (20%) datasets for the model development; within the training dataset, 10% was used for validation.

Generation of structural similarity profiles

A structural similarity profile (SSP) for an input drug was generated in the same manner as previously described(2), but with a greater number of approved drugs than the previous version DeepDDI: from 2,159 approved drugs to 2,386 approved drugs. A list of the approved drugs was obtained from DrugBank (1) (version 5.1.1). These approved drugs served as so-called 'fixed comparison targets'. Briefly, a SSP was generated by obtaining Tanimoto coefficient between an input drug and 2,386 approved drugs in a pairwise manner. Subsequently, two SSPs of each drug in an input pair were reduced to a dimension of 50 using principal component analysis, and merged to generate a feature vector, namely 'a combined SSP' that has 100 dimensions. The combined SSP was finally processed by a multilayer perceptron (MLP) of DeepDDI2 to predict DDI types for the input drug pair.

Development of a deep learning model for predicting DDI types

The updated DeepDDI2 has 113 output neurons, each of which represents a specific DDI type. Definitions of 113 DDI types are available in the form of sentences in Dataset S2. DeepDDI2 was designed to perform the multi-label classification where a drug pair can be assigned with

multiple DDI types. To train a DNN in DeepDDI2, weights of the DNNs were randomly initialized based on the uniform distribution. For activation functions, the rectified linear unit (ReLU) was used for hidden layers, while the sigmoid function was used for the output layer. The DNN was trained up to 300 epochs with early stopping set at 10 epochs, using binary cross-entropy as a loss function and Adam method (3) for optimization. In addition, the Monte Carlo (MC) dropout (4) was used to measure the prediction uncertainty (i.e., standard deviation) by generating ten MC dropout samples during the model training and for the new predictions. The mean and standard deviation of the ten prediction results are also generated as an output of the DeepDDI2.

Hyperparameters, including the number of hidden layers, the number of nodes per hidden layer, learning rate, and dropout rate, were optimized with respect to F1 score via Bayesian optimization (5) (Dataset S4). By examining 50 different architectures for the DNN, three hidden layers, 1,024 nodes for each layer, learning rate of 0.0069, and dropout rate of 0.3 showed the most optimal performance for the DeepDDI2. Threshold for an output neuron's activity (predictive mean - half a standard deviation) was set at 0.27; having this threshold resulted in the best F1 scores among the threshold values between 0 to 1 with an interval of 0.01.

Processing of the initial prediction results from the DNN

DDI types initially predicted by the DeepDDI2 were considered valid if they satisfy the following two criteria (6); 1) a drug should exist in the gold standard DDI dataset, which has a similar structure as one of the two input drugs (Tanimoto coefficient > 0.7); and 2) these structurally similar drugs from DeepDDI2 and the dataset should be associated with the same DDI type.

Preparation of a dataset for prescription drugs from FDA Adverse Event Reporting System (FAERS)

Of the 2,159,674 cases (prescriptions) reported in the FDA FAERS database from Q4 2012 to Q2 2018 (https://fis.fda.gov/extensions/FPD-QDE-FAERS/FPD-QDE-FAERS.html), only those involving two to five drugs, all assigned with DrugBank IDs, were considered in this study. As a result, 2,248 prescription drugs from the 301,615 FAERS cases were considered in this study. As a result of running the DeepDDI2, 1,628 prescription drugs were predicted to have DDIs with nirmatrelvir and/or ritonavir (Dataset S3).

Development environment

All the computations were implemented by using Python 3.6 in the Linux Ubuntu environment. A Python package Keras (version 2.0.6) (https://keras.io/) with the TensorFlow backend (7) (version 1.2.1) was used to develop the DNN of DeepDDI2.

SI References

1. D. S. Wishart *et al.*, DrugBank 5.0: a major update to the DrugBank database for 2018. *Nucleic Acids Res.* **46**, D1074-D1082 (2018).
2. J. Y. Ryu, H. U. Kim, S. Y. Lee, Deep learning improves prediction of drug-drug and drug-food interactions. *Proc. Natl. Acad. Sci. U S A* **115**, E4304-E4311 (2018).
3. D. P. Kingma, J. Ba, Adam: a method for stochastic optimization. *arXiv* (2014).
4. Y. Gal, Z. Ghahramani (2016) Dropout as a bayesian approximation: Representing model uncertainty in deep learning. in *Int. Conf. Mach. Learn.*, pp 1050-1059.
5. J. Snoek, H. Larochelle, R. P. Adams (2012) Practical bayesian optimization of machine learning algorithms. in *Adv. Neural Inf. Process. Syst.*, pp 2951-2959.
6. S. Vilar *et al.*, Similarity-based modeling in large-scale prediction of drug-drug interactions. *Nat. Protoc.* **9**, 2147-2163 (2014).
7. M. Abadi *et al.*, TensorFlow: a system for large-scale machine learning. *Proceedings of Osdi'16: 12th Usenix Symposium on Operating Systems Design and Implementation*, 265-283 (2016).
